# Digital onboarding in finance: a novel model and related cybersecurity risks

**DOI:** 10.12688/openreseurope.14289.1

**Published:** 2021-12-09

**Authors:** Miren Karmele García, Eliseo Venegas, SOTER, Esther Aguilera, José Manuel Panizo, Charlotte Kelly, Diego Serrano

**Affiliations:** 1 NTT DATA, Madrid, Spain; 2 Real Casa de la Moneda y Timbre, Madrid, Spain; 3 Accertify, London, UK; 4 Liberbank, Madrid, Spain

**Keywords:** Digital onboarding process, Cybersecurity, Customer identification, Finance sector, Trust.

## Abstract

Over the last few years, the financial sector has undergone a digital revolution that has had a severe impact on different related areas such as, the entities, the cybersecurity of systems, regulations and, of course, customers. SOTER project takes the challenge providing a complete set of tools to enhance the cybersecurity levels by implementing, in addition to non-technological tools, an innovative onboarding process has been implemented with the goals of increasing security, improving the user experience and integrity in the sector, and facilitating the customer entry into the digital marketplace by combining a set of breakthrough technologies. The cybersecurity research plays a crucial role in the conception and implementation of the onboarding process and for this kind of processes it has to be studied as an individual area by itself, as it is necessary to analyze the possible threats that can affect them, their origin and the solution(s) that can be taken to address them. Therefore, the SOTER project presents a fully digital onboarding process, innovative, adaptable to both current and future market needs and to possible changes in regulation, based on the most advanced technologies available today, and above all guaranteeing the cybersecurity of both entities and end users.

## Disclaimer

The views expressed in this article are those of the author(s). Publication in Open Research Europe does not imply endorsement of the European Commission.

## Introduction

In recent years, the financial sector has been experiencing a digital revolution in which traditional institutions have seen the need to become digital service providers in order to remain competitive.

This process of digital transformation is driving not only the development of new products and services, but also the progress and implementation of the very technology that supports them and which also enables innovation in the development of customer interaction with financial entities.

According to VASS data ^1^:

- 50 % of digital service applicants are under 35 years old.
- Six times a month, millennials access applications to manage their products.
- 70% of customers prefer to contract products digitally.

Nevertheless:

- Only 8% of the applications allow the process to start and finish on the mobile device.
- 60% of all hires involve attending a branch.

Until recently and according to legislation, it was necessary to go in person to register as a customer in order to verify the customer's identity. Nowadays, in response to this growing demand, financial institutions have the technical means to identify their customers in a non-face-to-face manner, so the sector is exponentially expanding its possibilities to operate remotely.

It is, also, necessary to take into account the current context of the COVID-19 pandemic we are experiencing since the last months of 2019, which has led to a reduction in the availability of services in physical offices, as well as limitations in terms of people's mobility as a result of social confinement.

As Forbes publishes ^2^, limitations caused by the lockdown have pushed companies to shift their resources toward digitalization. According to Efma and Infosys Finacle's latest "Innovation in Retail Banking" report ^3^, 75% of financial organizations name digital banking transformation as their top priority for 2021, followed by customer experience improvements (51%). Also, now that we are forced to restrict social contact, customers who were sometimes reluctant to use such processes have not only ended up integrating technology naturally into their relationship with the bank but are even beginning to actively demand it.

On the other hand, regulators and participants in the financial industry, whose main function is to take care of the identification of customers and, therefore, to protect their assets with mechanisms that guarantee the integrity and security of their assets, avoiding risks of fraud and money laundering, are, somehow, obliged to change their mentality in order to build a new legal framework.

For all the participants, digital onboarding processes bring great benefits to transform the paradigm of remote customer acquisition, which previously only happened within a traditional face-to-face banking scheme. Today, technological innovation in the financial industry means that this banking model is being revised and there is an opportunity to acquire users and sell products outside the physical space through digital channels.

## SOTER project

SOTER project adopts a holistic research approach that combines two complementary avenues of research (technical and non-technical) to address the increasingly complex nature of cybersecurity resilience. The first line of research, and the subject of this article, focuses on the development of a biometric-based digital identity and authentication onboarding platform for the financial services sector. This technological tool aims to increase security, ease of use and integrity in the sector, facilitating customer entry into the digital marketplace by combining a set of breakthrough technologies.

The platform is also being built according to privacy by design, security by design, data protection by design and default methodologies which will seek to ensure transparency and accountability throughout the design and development lifecycle. It will provide a series of public reports and deliverables communicating to the public how it has adhered to the applicable regulatory frameworks (GDPR, eIDAS, 5AMLD, PSD2, etc.), as well as exposing to the general public how privacy and data protection principles are upheld, in line with European Commission guidelines.

### Digital onboarding processes: background ^4^

A digital onboarding process is the non-face-to-face identification process that allows users to register as new customers and remotely open financial products and services 100% digitally through online channels. The customer does not need to physically visit the bank or complete forms, and the process can be carried out through any digital device. Identification and registration are based on the use of biometric technologies for recognition and the capture of official identification documents, while complying with the applicable rules and regulations imposed by the competent organisms in the field. All the information collected during the process is stored and used for an adequate manual validation of the applicant's data, and thereby facilitating access to the service or product required.

Undoubtedly, this is a process that is as revolutionary for the financial environment as it is complex to implement, since the strict regulations governing it, such as the obligation to verify the customer's identity physically and another series of legal requirements aimed at preventing financial crimes such as money laundering or the financing of criminal activities, did not allow it to be done remotely until recently.

***Evolution of customer registration models.*** Having successfully mastered the revolution that the financial sector has been experiencing the past recent years in which traditional entities are becoming providers of digital services in order to remain competitive, these entities are now building their future on the technological pillar that allows them not only to offer new products and services, but also new customer relationship models.

But before even considering these types of processes, the main approaches offered by financial entities for registering customers are as follows:

- Face-to-face model: This is, traditionally, the most popular model. It requires the customer to physically go to the office to fill in the documentation and sign the relevant contracts, having previously demonstrated their identification documents to the employee in order to prove their identity in situ.
- Semi-digital model: The customer can download the forms and contracts online to fill them in and sign them manually whenever he/she deems appropriate. Afterwards, however, the user will have to go to the office to identify him/herself and present the documents. The advantage of this model is mainly limited to the possibility of filling in the forms from any location, but it does not avoid the need to travel, unless the institution itself provides the service of sending a postman to the address indicated by the customer so that he/she can identify him/herself, as many institutions have already done.
- The entry into legal effect of the Sepblac regulation as of 1 March 2016 allows the use of non-face-to-face identification procedures via video conferencing. This means that new customers can be recruited online, albeit subject to certain requirements.
- This regulation gave origin to the digital onboarding model, or non-face-to-face identification by video call, which allows a customer to register with a financial institution by showing a supporting identity document in front of the camera of a computer or mobile device. This model offers an immediacy and convenience never seen before in the sector, while at the same time complying with the legal requirements imposed by regulatory bodies.
- Digital model: By providing the identifying information of an account (IBAN) at any Spanish bank of which he/she is already the account holder, the customer can apply for affiliation with any other bank. If the response is positive - as is usually the case - the process will be 100% digital. The customer will not need to interact with any other person and will be fully authorized to operate. SEPA transfers, which have been authorized since June 2016, provide the necessary data to verify the user's identity.

### SOTER project digital onboarding process

everis Spain, as main technological partner of SOTER project has designed a digital onboarding process intended to be a technological tool that facilitates the interconnections between the different service providers (EADS, InAuth, Trunomi and FNMT) and the users in a simple way. It provides identification and onboarding in a Software as a Service model to allow the delivery of electronic transactions at any time, in a transparent way, to any kind of service provider belonging to any market sector.

everis Spain has developed a platform which acts as an independent third party that enables identification for making electronic transactions in a decentralized way from the final services. This approach allows the owner of the final services to focus on their core business and to detach himself from investing on creating such identification services compliant with regulations because SOTER platform will provide ready for them.

***Digital onboarding process’ infrastructure.*** everis Spain as experts in leading edge technology, propose an infrastructure which intends to reach the goal of creating a platform able to create digital cores on different markets. This infrastructure is composed by the architecture and a mobile library.

The architecture is the backbone of the onboarding process, managing every step of it and the integration and communication with third parties. In order to be able to fulfil its tasks, the following approaches are being used:

- It is a cloud native architecture, which means that is developed using cloud-based technologies.
- APIfication business model is used because it makes the architecture more flexible and productive.
- Microservices technology is used as they are small independent services responsible for implementing full business functionality.

On the other hand, everis Spain has developed an app which can be easily integrated in the entity’s main mobile application. This way, the future client does not have to download an additional app on his device and the entity can invoke the digital onboarding process from the points of its mobile app as it deems appropriate. At a high level, the app is the front-end of the digital onboarding process as it interacts with the clients and plays the role of an intermediary interpreting the customer interactions with the screens of the onboarding process and transferring them to the architecture to meet the customer’s requests.

The most relevant functionalities included in the onboarding process are described below.

***Identification process.*** everis Group’s Aerospace and Defense Division (EADS) offers global solutions for implementing critical systems in aerospace, defense, security, and simulation sectors, integrating reliable and innovative technologies though proprietary development. Specifically for the digital onboarding process implemented at SOTER, they collaborate by providing their identification and identity verification services whose capabilities contribute to making the process faster and 100% digital for the entity's customers.

The technology provided by EADS is designed to complete the document verification and confirm that it corresponds to the user on the other side of the device performing the onboarding process. The solution ensures that the person presenting the identity is the rightful owner and is a live person who is physically present at the time of identification, eliminating the need to send additional documentation and making the process more streamlined for both the customer and the entity.

It is important to assess the performance and security of any identification system in order to identify and protect against threats, attacks, and exploitable vulnerabilities. Security breaches are, most commonly, the result of an exploited vulnerability. This includes poor physical security which continues to be an easily exploitable attack vector. The onboarding process will face a holistic approach in its biometric system, more precisely, in the digital onboarding process, the state-of-the-art of EADS technology is based on the facial recognition system. Although there are several types of biometric captures that can be performed, some of them already known like the fingerprint ^5^ and some other more atypical ones like wrist vein images ^6– 8^ or Finger-Knuckle-Print (FKP) ^5, 9^ recognition, the facial biometrics has drawn significant attention due to its potential use in biometric authentication ^10^ as non-intrusive and simple way to capture the facial image of the customer.

The challenge regarding facial biometrics is to create the biometric pattern for future recognition processes in a flexible way. In addition, it is very important to generate a biometric pattern from a real person, preventing any kind of human representation such as pictures, videos, masks, etc. in the digital onboarding, preventing any kind of fraud in the registration process. Among the functionalities provided by EADS is the ability provide anti-spoofing proof of live based on different capabilities such as blinking, random and unpredictable movements or behavioral traits (i.e. smile). For the project, they propose a solution that combines all previous capabilities and technologies at the same time, providing a robust anti-spoofing ^11^ solution for digital onboarding.

***End-to-end privacy compliance and management.*** Trunomi provides the Data Privacy compliance layer for the SOTER onboarding platform, providing Privacy services for end-users, as well as the easy evidencing and management of compliance for the banks utilizing SOTER platform.

During the onboarding process, Trunomi provides a seamless experience for end-users by sitting behind SOTER’s UI. As consent is requested and data is collected through the interface, Trunomi’s APIs generate granular records of data processing that are stored on the SOTER platform, providing transparency as to how end-user data is processed.

For the bank, Trunomi enables compliance with the highest standards of Data Privacy during end-user onboarding. As well as providing the bank with trusted intelligence for audits, secure, tamper-proof and queryable records of data processing can be shared with third parties and can be used to trigger workflows with any downstream systems. Privacy intelligence is automated via Reporting, where they can understand their end-users’ PII, data permissions and processing activities from a single screen.

***Device identification.*** Accertify’s main role within SOTER’s onboarding platform is to provide device intelligence information to identify devices which contributes to the overall verification and fraud prevention strategy.

Device Intelligence is powered by Accertify and prevents fraud by analyzing devices and associated identities transacting across digital channels via mobile applications. Accertify’s device platform helps clients verify identity, assess, and mitigate risk in real-time, and optimize the customer experience.

Accertify’s InMobile product provides a Device Intelligence Software Development Kit (SDK) that can be incorporated into mobile applications to access mobile device information. Device information and operating system attributes can be collected and analyzed to produce a persistent device identifier that prevents fraud. It is therefore resilient to tampering, application uninstall/reinstall, and OS upgrade.

As part of contributing to SOTER’s fraud prevention strategy, InMobile analyses connected devices to detect known malicious applications as well as criminal tools such as location spoofing and IP address proxy apps. Malware files are dynamically updated without client interaction. InMobile also protects against complex rooting methods used by fraudsters, such as cloaked Root, through to Advanced Root and Jailbreak Detection. Further, InMobile’s security architecture, “Trusted Path”, prevents interceptions by providing a secure path to transport sensitive information and messages. It is encrypted end-to-end, signed, and digitally protected against replay attacks.

The SOTER Digital Onboarding Platform will also be utilizing the InRisk product. InRisk is a device data analysis and risk assessment system with configurable business rules and device scoring that allows the deployer to measure device trustworthiness. InRisk provides a set of standard rules that analyze data attributes of mobile devices to identify unusual or suspicious behavior indicating potential risk. As risk tolerances vary, customers are provided the flexibility to tailor thresholds and scoring to meet their specific needs.

***Trust service provider.*** FNMT-RCM will play a key role by making legally valid the transactions accomplished in the onboarding process. For that purpose, FNMT-RCM will offer a set of electronic services that are explained in the following sections.

### e-Signature

After gathering all the required information, the client will be asked to sign a service contract, which will attest the user acceptance of the service conditions offered by the financial institution. Therefore, in its activity as a Trust Service Provider, FNMT-RCM provides a centralized signature tool.

FNMT-RCM signature service will create advanced electronic signatures which will meet the requirements set out in the article 26 of eIDAS regulation:

An advanced electronic signature shall meet the following requirements:

- it is uniquely linked to the signatory;
- it is capable of identifying the signatory;
- it is created using electronic signature creation data that the signatory can, with a high level of confidence, use under his sole control; and
- it is linked to the data signed therewith in such a way that any subsequent change in the data is detectable.

In an earlier phase of the onboarding process, the certificate holder (the customer) must be informed that FNMT-RCM is issuing a certificate on their behalf and also, that this certificate and its relevant key will be used to sign the service contract on their behalf.

### Timestamping

A trusted timestamping service provides assertions of proof that an information existed before a particular time. If this service is managed by a Time Stamp Authority (TSA), it embodies an independent, reliable, and irrefutable source. Therefore, it must be used as a process to securely keep track of the creation and modification of a document, a transaction, or a digital signature. Among the objectives to be achieved in a secure system, a timestamping service helps to procure integrity and authenticity: every change applied to a piece of information will be detected and a trusted third party assures the reliability of the act of trust. In legal terms, it creates strong evidences that a business transaction happened at a particular moment in time, or a contract between two identified parties was created and nobody has modified it since then. A timestamping token can be digitally stored, and it can be validated in the long-term.

FNM-RCM is a qualified trust services provider of a timestamp service. The term qualified is expressed within eIDAS Regulation, which sets a legislative framework for the development of electronic services in a context of cross-border digital markets. The main advantage of a qualified trust service provider is the fact that it has met a set of requirements and it has been checked and audited by a European-level supervisory body to enable its activity. A qualified trust service offers legal and technical security guarantees in electronic transactions. In addition, the private keys that signs the timestamp are protected within a qualified electronic signature creation device, which fulfils the requirements of eIDAS Regulation Anex II. This regulation also expresses the requirements for qualified electronic timestamps which are:

- it binds the date and time to data in such a manner as to reasonably preclude the possibility of the data being changed undetectably;
- it is based on an accurate time source linked to Coordinated Universal Time;
- it is signed using an advanced electronic signature or sealed with an advanced electronic seal of the qualified trust service provider, or by some equivalent method.

### Electronic custody

This service is a response to one of the strengths of SOTER Digital Onboarding platform, which is the provision of legal validity to any kind of electronic transaction performed by the platform, is not designed or intended to be used as a user cloud storage. The integration of a trusted third party, such as FNMT-RCM, will certify and custody the required evidences.

FNMT-RCM, as its role of custodian of digital assets, will offer a high level of security for the preservation of the evidences that are collected during the onboarding process. They will be stored in an Enterprise Content Management tool (ECM), which is licensed by FNMT-RCM and is deployed locally on its own servers in an on-premise security data center. An ECM server goes beyond a traditional document archive. It enables record management capabilities, including metadata schemas, record retention policies, safe deletion, safekeeping, electronic signatures, or electronic seals.

FNMT-RCM’s ECM tool is able to design a rich data model and thus, opens up new possibilities to store complex elements such as compound documents which is an abstraction to represent multiples documents or evidences as a whole. FNMT-RCM’s custody service will be audited in order to assess its security.

## Cybersecurity risks in digital onboarding processes

Since the use of onboarding processes, cybersecurity in this type of process needs to be studied as an area in its own right. This section will set the foundations for this study, making a taxonomy of the threats that can affect this type of process from a generic point of view.

To be able to offer a state-of-the-art security platform, it is necessary to understand, research and analyze the threats that can affect the onboarding process, their origin and the solution(s) that can be taken to address them. To achieve this goal, security needs be considered in a holistic approach, being studied, and considered both in the process and in the IT platform on which this process takes place.

The digital onboarding process is closely related to the Regulation (EU) No 910/2014, which “ensure that secure electronic identification and authentication can be used to access cross-border online services offered by Member States.” ^12^

According to ENISA, there are some tendencies and considerations that should be pointed out in order to study the security of onboarding processes and platforms:

- The increasing usage of mobile-based systems on onboarding process.
- Use of multimodal biometric systems and behavioral biometrics (multiple sensors, samples, traits, instances, or representations).
- The growing involvement of the private sector.
- Management of their Personal Identifiable Data by users and concern for user privacy and data protection.

The cybersecurity analysis of the onboarding process is divided into two steps: the threat landscape identification and analysis and the proposal of security measures for avoiding identified threats.

### Threat identification and analisys

In order to identify the threats that may affect the onboarding process, a research of the context in which the process takes place is carried out, developing a threat landscape which covers all the threats that can come from the banking sector, the cloud computing systems, and the Third-Party ecosystem. These topics encompass all the context in which the onboarding process is framed. The main goal is to focus on securing the digital onboarding process platform as a whole.

ENISA proposes some security considerations ^12^ on which documents and standards should be taking into account when securing the Onboarding process and platform:

- Commission Implementing Regulation 2015/1502 (Article 8(3) of the eIDAS Regulation) ^13^.
- ISO/IEC 29003:2018 – Identity proofing ^14^.
- ISO/IEC 29115:2013 – Entity authentication assurance framework ^15^.
- NIST SP 800-63 – Digital Identity Guidelines ^16^.
- CEN/TR 419010 – Framework for standardization of signature – extended structure including electronic identification and authentication ^17^.

In following subsections, main threats that can affect the onboarding platform will be outlined targeting on the following topics: Banking sector, Cloud computing and Third-Parties data sharing implicit risks.

Finally, a section is added proposing the identified security measures that are best suited to the onboarding platform and mitigate the risks arising from the threats presented.

***Threats inherited from the Banking Sector.*** The complexity of the financial sector makes it hard to interpret the threat landscape, as different domains within financial services and banking may face entirely different cyber risks and threats. However, ENISA has conducted a study on the most common attacks faced by this sector ^18^:

***Web application attacks.*** Due to the increased use of web applications in all sectors, these applications and technologies have become a core part of the internet, and therefore, more attractive to cybercriminals, making attacks against these applications more frequent nowadays.

To mitigate the risks arising from potential threats or attacks against these applications and technologies, the following initiatives are proposed:

1. Involvement of a cybersecurity team in the development of the applications to ensure the correct implementation of security features in the web application.
2. Securing APIs by implementing authentication measures, authorization measures, connection logs or connection encryption among other measures ^19^.
3. Improving authorization methods to avoid data theft.
4. Use input validation and isolation techniques to avoid code injection attacks. Two out of three attacks to web applications are made by SQL injection and finance sector is more vulnerable to Local File inclusion attacks. Data from security researches ^20– 22^ suggest that over 90% of web attacks are based on SQL injection and Local File inclusion attacks.

***Insider threat (unintentional abuse).*** This threat domain refers to all those mistakes that employees make ^23^, due to lack of knowledge or carelessness. Most of these attacks consist of phishing attempts, due to spear phishing attacks, poor passwords, orphaned accounts or browsing suspicious sites.

To avoid these attacks, it is recommended to introduce an insider threat countermeasures plan into the overall security strategy and policies and to draw up a security policy on insider threats, based on user awareness ^24^.

***Malware.*** There are many types of malware that can affect the banking sector, and most are delivered via e-mail and spread by employees’ activity ^25^. The most prominent malware from an internal perspective is the following:

- Malware as a Service (MaaS): Specific malware sold in underground forums.
- Mobile banking malware, like banking Trojans.
- File-less malware, which do not contain an executable file and can easily evade common security filters and whitelisting techniques. The most important are Script-based attacks, In-memory attacks, and System built-in tools ^26^.
- Botnet and Command and Control malware: Malware that controls one or more Internet-connected devices to perform DDoS-type attacks on other systems.

There are many measures to prevent the spread of malware, but the following are some basic measures that may dramatically reduce the risk of infection: it is recommended to implement malware detection for all channels, to inspect SSL/TLS traffic, employ mail filtering and use malware analysis tools ^27^.

***Data theft.*** Data breaches are a very dangerous threat, as they can result in costs that last long after the incident has occurred and can be caused by small mistakes, leading to large breaches.

Threat actors usually focus on financial data when they perform a data theft.

The most common attacks to carry out data breaches are:

- Phishing, focusing on third-parties or partners e-mails.
- Cloud/Web application attacks, stealing credentials to access to web-based e-mail portals or exploiting weaknesses in application servers.
- Insider errors/Malicious insider, unauthorized or malicious attempts to use resources. Most data breaches are facilitated by external malicious actors exploiting insider errors.

To protect the systems, it is recommended to apply the following actions among others: establish and maintain an incident response team and plan, encrypt sensitive/personal data, and develop and maintain strong passwords policies.

Data breaches are often the result of other threats, so it is important to conduct a risk assessment to identify and protect systems from these threats.

***Threats inherited from Cloud Computing systems.*** The use of cloud computing for the deployment of applications and systems is growing continuously due to the advantages of working with cloud services.

But the use of cloud computing systems brings not only advantages, but also very specific security threats ^28^ due to the architecture involved in the use of cloud computing:

***Software vulnerabilities.*** Cloud computing systems are especially vulnerable against software vulnerabilities.

A specific vulnerability of software type that only occurs in cloud systems is the isolation failure, which means that one customer access to another customer’s data.

For avoiding software vulnerabilities, it is useful the implementation of advanced process to develop, deploy and maintain software.

***Network attacks.*** This threat includes attacks like spoofing websites, eavesdropping network traffic or Denial-of-Service attacks ^29^ among others. To avoid these risks, it is recommended to implement network protection like firewalls, records of activity and/or deploy a demilitarized zone (DMZ), among others.

***Social engineering.*** A social engineering attack consist in an attacker faking communication or information, so it appears to come from a trusted source. This attack is focused on every actor that interacts with the cloud system.

The way to prevent social engineering attacks is to raise awareness among cloud platform participants.

***Interface management compromise.*** Customers should verify that the interfaces to connect to the cloud systems use good authentication mechanisms. Also, security of PCs connecting to a cloud platform should be taken into account.

There are multiple measures that can be addressed to avoid the interface compromise like authentication measures, authorization measures or connection logs among other measures.

***Device theft or loss.*** Some devices that usually connect to the cloud system are mobile devices, which are relatively vulnerable to theft and loss, which could mean the theft of data or authentication credentials.

This can be addressed by implementing measures such as screenlocks, media encryption and/or providing corporate devices to employees (measure for companies).

***Overloads.*** In case a large number of users want to access the platform, or the platform is affected by a DDoS attack, the vulnerability of the cloud platform could be affected. This could be solved by developing a proactive and strong defense against critical network failures, configuring cache servers, or dropping inappropriate requests and implementing a BCP. Having a clear channel and plan of communication between internet provider and Cloud service providers is also crucial.

***Vendor lock-in.*** Is the case when is hard to migrate to another cloud provider. To avoid this threat, it is recommended to back up data regularly and to develop business continuity strategies, which includes migrate plans.

***Threats inherited from Third-Parties ecosystem.*** The Third-Parties ecosystem is a very large area that depends very much on the specific solution in question ^30^.

Due to its nature, the banking/financial sector tends to depend on service providers more than other sectors. Many applications or systems, such as cloud solutions, rely on providers to supply them with services that are essential for their correct and complete operation.

Therefore, this ecosystem must be analyzed in order to understand what kind of impact these providers can have on the Onboarding process.

Following are most critical threats when sharing data with Third Parties.

1. Data breaches, produced by vendors.
2. Third party governance programs, many companies need to improve the managing outsourced relationship risks.
3. Lack of visibility, companies should have an inventory of which data are sharing with which service provider.
4. Poor third-party risk management, companies should ensure that third parties have the necessary safeguards and security policies in place to prevent security incidents.

## Conclusions

The onboarding process proposed by SOTER project is the outcome of the need that emerged as a result of the digital progress experienced in recent times and as the last step of the path that has been taken in the area of customer acquisition in financial institutions.

This innovative and flexible process is the result of the work of several partners who have contributed their products and efforts to obtain a platform that tries to contemplate all aspects in a way that provides a completely digital solution that allows the potential client to carry out all the steps of the process from beginning to end in an agile, simple and totally cybersecure way for all the actors involved. At the same time, it is totally flexible and configurable to adapt to the particularities of the client entities, to possible changes in regulations and even to new technologies that may come onto the market in the future, and with the guarantee that the platform is competitive from the commercial perspective.

It is important to highlight that the cybersecurity aspect is essential for the onboarding process, as it is key to ensure that the entire process is secure. The security of the platform security must be addressed as a whole, applying security controls to technology solutions and relying on secure policies and procedures to provide a complete security picture, avoiding vulnerabilities and security incidents.

In a process, such as onboarding, which involves different separate components operating in completely different environments, it is required that all components and their environments be secure in order to maintain the level of security throughout the process.

For this purpose, the following recommedations are proposed to ensure the confidentiality, integrity and availability of the data and services related to the onboarding process.

### Awareness

As mentioned previously, the banking sector is highly exposed to attacks targeting employees and users, such as phishing and scams. That is why it is of great importance to train and provide security awareness training to both the employees involved in the process and the end users of the platform.

### Contractual Agreements

When working with third parties, it is of critical importance to establish contracts and agreements that define the role of each actor and define permitted and forbidden actions.

This is also very important when it comes to cyber security. When sharing information and services with third parties, agreements must be established that specify the minimum level of security required and how this level of security is to be achieved.

### Business Continuity

Preventing business disruption and minimizing business impact is a major concern of cyber security. For this purpose, it is recommended to define several procedures and controls related to the continuity of activities like defining a well-structured Business continuity Plan, defining a detailed Contingency plan and make periodic System Backups.

### Data and system integrity

As discussed above, malware and exploiting flaws in the software that supports the onboarding process is one of the biggest issues that can damage the data and services involved in the onboarding process. It is therefore highly recommended to deploy measures to protect against malicious code and to detect flaws in order to solve incidents as soon as possible.

### Identification and authentication

Securing the process starts with ensuring correct and reliable authentication of the user by means of hardware authentication and confirmation of the user's identity by means of biometric testing. This can be ensured by implementing strong authentication procedures and cryptographic measures like Two Factor Authentications, secure key management and key and data encryption.

### Risk Assessment

The Risk Assessment is a very useful method to determine the security status of a system and to identify the main vulnerabilities that need be addressed in a system. This process is periodic and should be reviewed, at least, once every year or every two years.

### Incident response

If an attack is successful, a security incident occurs on the platform. These incidents are usually detected manually by workers who detect some anomalies in the onboarding process or automatically by specific software called SIEMs (Security information and event management). It is imperative that security incidents are addressed with according to the established Incident Management Plan and reported to the relevant authority in time.

All security incidents should be internally recorded and tracked, in all their stages, until they are fully resolved and closed.

Specific security controls can be found in the ISO/IEC 27001:2013 standard ^31^, the NIST SP 800-53 controls ^32^ and the Directive (EU) 2016/1148 ^33^.

## Data availability

No data are associated with this article.
