# Supplementary material for: Digital onboarding in finance: a novel model and related cybersecurity risks
Source: Open Res Eur. 2022 Mar 22;1:149. Originally published 2021 Dec 9. [Version 2] doi: 10.12688/openreseurope.14289.2 (PMC10446084; doi:10.12688/openreseurope.14289.2)
Supplement: Peer review history

# Peer review: Reviewer response for version 2

**DOI:** 10.21956/openreseurope.15685.r28898

**Published:** 2022-04-12

**Authors:** Dariusz Grabara

**Affiliations:** 1 University of Economics in Katowice, Katowice, Poland

Thank you for your response. Suggested issues have been addressed. However, it would be great to add information about PUF, and shortened information about certificate process (for example as footnotes) to the original article. Unfortunately, cybersecurity risks are major concerns nowadays.

Where applicable, are recommendations and next steps explained clearly for others to follow? (Please consider whether others in the research community would be able to implement guidelines or recommendations and/or constructively engage in the debate)

Partly

Does the article adequately reference differing views and opinions?

No

Are all factual statements correct, and are statements and arguments made adequately supported by citations?

Partly

Is the rationale for the Open Letter provided in sufficient detail? (Please consider whether existing challenges in the field are outlined clearly and whether the purpose of the letter is explained)

Partly

Is the Open Letter written in accessible language? (Please consider whether all subject-specific terms, concepts and abbreviations are explained)

Yes

Reviewer Expertise:

NA

I confirm that I have read this submission and believe that I have an appropriate level of expertise to confirm that it is of an acceptable scientific standard.

# Peer review: Reviewer response for version 2

**DOI:** 10.21956/openreseurope.15685.r28899

**Published:** 2022-03-29

**Authors:** Pietro Tedeschi

**Affiliations:** 1 Technology Innovation Institute, Secure Systems Research Center, Abu Dhabi, United Arab Emirates

The authors addressed most of the comments in this revised version.

 Please about the reference, you can cite for example: https://www.sciencedirect.com/science/article/abs/pii/S135348582100088X

Where applicable, are recommendations and next steps explained clearly for others to follow? (Please consider whether others in the research community would be able to implement guidelines or recommendations and/or constructively engage in the debate)

Partly

Does the article adequately reference differing views and opinions?

Yes

Are all factual statements correct, and are statements and arguments made adequately supported by citations?

Partly

Is the rationale for the Open Letter provided in sufficient detail? (Please consider whether existing challenges in the field are outlined clearly and whether the purpose of the letter is explained)

Partly

Is the Open Letter written in accessible language? (Please consider whether all subject-specific terms, concepts and abbreviations are explained)

Yes

Reviewer Expertise:

Unmanned Aerial Vehicles Security, Maritime Security, Wireless Security, Internet of Things (IoT), Applied Cryptography, Privacy Preserving Systems, Critical Infrastructure Security, and Cyber-Physical Systems Security

I confirm that I have read this submission and believe that I have an appropriate level of expertise to confirm that it is of an acceptable scientific standard.

# Peer review: Reviewer response for version 1

**DOI:** 10.21956/openreseurope.15417.r28412

**Published:** 2022-02-04

**Authors:** Dariusz Grabara

**Affiliations:** 1 University of Economics in Katowice, Katowice, Poland

The paper is focused on digital onboarding of the customer of financial institutions in the context of SOTER project. The paper present steps taken for customer onboarding in relation to cybersecurity risk mitigation. The framework is discussed with examples of implementation on several finance institutions. The paper is well organized, however additional concerns should be addressed. Concerns are as follows:

- Author states: "It is, also, necessary to take into account the current context of the COVID-19 pandemic we are experiencing since the last months of 2019…”. Please state the source of this sentence since in Europe COVID-19 first confirmed case was dated on 24 January 2020 (see doi:10.2807/1560-7917.ES.2020.25.6.2000094). SOTER consortium is drawn from European member states and UK.
- Author states that “It will provide a series of public reports and deliverables communicating to the public how it has adhered to the applicable regulatory frameworks (GDPR, eIDAS, 5AMLD, PSD2, etc.), as well as exposing to the general public how privacy and data protection principles are upheld, in line with European Commission guidelines.” It should be mentioned that the basis for the regulations is lying in Article 13(1) of Directive (EU) 2015/849.
- Author states about digital onboarding: “Undoubtedly, this is a process that is as revolutionary for the financial environment as it is complex to implement.” The process was known to the financial sector. The European Banking Authority (EBA) has developed onboarding guidelines and consultation paper has been issued on 10 ^th^ December 2021. Look at EBA/CP/2021/40 (link: https://www.eba.europa.eu/eba-consults-new-remote-customer-onboarding-guidelines). It should be also mentioned in the presented paper, that process has been pointed out by the European Commission in Digital Finance Strategy for the EU in a communique from 24 ^th^ September 2020 (link: https://eur-lex.europa.eu/legal-content/EN/TXT/?uri=CELEX:52020DC0591). While in some cases process could be perceived as revolutionary overall it is known to the financial sector and implementations are already ongoing. In that case, it would be interesting for readers to provide references of the SOTER project in the perspective of the EBA Consultation Paper.
- Author specifies that one digital model for registering customers is by providing affiliation from any other Spanish bank. Please add information that this example is not limited to Spain only but also used worldwide.
- Author states: “Among the functionalities provided by EADS is the ability provide anti-spoofing proof of live based on different capabilities such as blinking, random and unpredictable movements or behavioral traits (i.e. smile).”. What is the effectiveness of this solution? What type of criteria do they meet to be considered reliable? The identification part of digital onboarding is very crucial. Proposing biometrical identification in the financial sector which could be omitted in some cases (for example face identification from iPhones) should be addressed.
- Author states in the "End-to-end privacy compliance and management" section: “For the bank, Trunomi enables compliance with the highest standards of Data Privacy during end-user onboarding.”. The phrase “highest standards” should be explained. What financial standards do the Trunomi meet? Please provide one from the perspective of EU regulations.
- In the “Device identification” section Author states: “Device information and operating system attributes can be collected and analyzed to produce a persistent device identifier that prevents fraud. It is therefore resilient to tampering, application uninstall/reinstall, and OS upgrade.” What type of resilience is Author referring to? Do they use physical unclonable functions (PUF)?
- Authors state that “FNMT-RCM’s custody service will be audited in order to assess its security.” Which institution will perform the audit?
- Author state about “threats inherited form Bankich Sector” that “ENISA has conducted a study on the most common attacks faced by this sector”. The SOTERO project should be able to work in different countries. Do ENISA study account for different countries?
- In “Conclusion” section Author states: “In a process, such as onboarding, which involves different separate components operating in completely different environments, it is required that all components and their environments be secure in order to maintain the level of security throughout the process.”. Different components need to operate on one platform. SOTERO integrates those services. However, the integration platform should also be audited not by only sum of audits but as a whole platform. What were the integration tests chosen for the platform?

Where applicable, are recommendations and next steps explained clearly for others to follow? (Please consider whether others in the research community would be able to implement guidelines or recommendations and/or constructively engage in the debate)

Partly

Does the article adequately reference differing views and opinions?

No

Are all factual statements correct, and are statements and arguments made adequately supported by citations?

Partly

Is the rationale for the Open Letter provided in sufficient detail? (Please consider whether existing challenges in the field are outlined clearly and whether the purpose of the letter is explained)

Partly

Is the Open Letter written in accessible language? (Please consider whether all subject-specific terms, concepts and abbreviations are explained)

Yes

Reviewer Expertise:

Online auction platforms, Industry 4.0, Artificial Intelligence (AI), E-Government services maturity, Enterprises cybersecurity.

I confirm that I have read this submission and believe that I have an appropriate level of expertise to confirm that it is of an acceptable scientific standard, however I have significant reservations, as outlined above.

## References

[1] : First cases of coronavirus disease 2019 (COVID-19) in France: surveillance, investigations and control measures, January 2020. *Euro Surveill* .25(6) : 10.2807/1560-7917.ES.2020.25.6.2000094 10.2807/1560-7917.ES.2020.25.6.2000094 32070465PMC7029452

# Response

**Published:** 2022-02-08

**Authors:** Esther Aguilera

**Affiliations:** NTT DATA, Spain

Hello, I am replying to some of your comments that I felt were not necessary to be included in the document.

- Regarding the EADS issue: this solution is implemented in places that require maximum security, such as police stations and airports. It complies with eIDAS and AML regulations.
- Regarding the "Device identification" section, yes, Accertify uses PUF, regarding resilience it refers to the fact that if the user installs or uninstalls the app or if he/she changes device, Accertify's intelligence is able to continue identifying whether or not it is the same user accessing the app.
- Regarding the FNMT-RCM's audits:
  - The certificate issuing system undergoes an annual audit process in accordance with the ETSI EN 319 401 "General Policy Requirements for Trust Service Providers" and ETSI EN 319 401 "General Policy Requirements for Trust Service Providers".Service Providers" and ETSI EN 319 411-1 "Policy and security requirements for Trust ServiceProviders issuing certificates".
  - Likewise, the Certificates that are considered as qualified, are subject to an annual audit that guarantees annual audit that guarantees compliance with the requirements established in the European standards ETSI EN 319 411-1 "Policy and security requirements for Trust Service Providers issuing certificates". standards ETSI EN 319 411-2 "Requirements for trust service providers issuing EU qualified certificates".
  - The Qualified Time-Stamping Service offered by the FNMT-RCM is subject to annual audits, according to the certification scheme corresponding to the Trust Service Providers, as to the Trust Service Providers, in terms of compliance with the requirements defined by the European standards ETSI EN 319 European standards ETSI EN 319 401 "General Policy Requirements for Trust Service Providers", ETSI EN 319 421 "Trust Service Providers issuing Time-Stamps" and ETSI EN 319 422 "Time-stamping protocol and time-stamp token profiles".
  - The server signature service offered by the FNMT-RCM is subject to annual audits, according to the certification scheme corresponding to the Trusted Service Providers, in terms of compliance with the following requirements Trust Service Providers, in terms of compliance with the requirements defined by the European standard ETSI EN 319 401 "General Policy Requirements for Trust Service Providers".
  - The Certificate issuance system is subject to additional audits:
    - Audit of the Information Security Management System according to UNEISO/IEC 27001 "Information Security Management Systems (ISMS). Requirements".
    - Audit as dictated in the National Security Scheme (Royal Decree 3/2010, of January 8, 2010, of3/2010, of January 8, 2010, which regulates the National Security Scheme in the field of Electronic Administration).
    - Audit of the Quality Management System according to ISO 9001.
    - Audit of the Social Responsibility Management System in accordance with IQNet SR10
    - Audit of the Business Continuity Plan according to ISO 22301.
    - Audit in accordance with Regulation (EU) 2016/679 of the European Parliament and of the Council of 27 April 2016 on the protection of individuals with regard to the processing of personal data and on the with regard to the processing of personal data and on the free movement of such data and repealing Directive 95/46/EC, and the Organic Law 3/2018, of December 5, 2018, on the Protection of Personal Data. December 5, on the Protection of Personal Data and guaranteeing the protection of personal data.
- Regarding ENISA framework, yes, it has studied accounts for different countries. You can find more information here https://www.researchgate.net/publication/334853829_The_new_EU_cybersecurity_framework_The_NIS_Directive_ENISA's_role_and_the_General_Data_Protection_Regulation
- The chosen test to audit the platform has been pentesting carry out by NTTDATA cybersecurity team. https://www.enisa.europa.eu/publications/digital-identity-leveraging-the-ssi-concept-to-build-trust

Thank you so much and best regards, Esther

# Peer review: Reviewer response for version 1

**DOI:** 10.21956/openreseurope.15417.r28386

**Published:** 2022-01-27

**Authors:** Pietro Tedeschi

**Affiliations:** 1 Technology Innovation Institute, Secure Systems Research Center, Abu Dhabi, United Arab Emirates

The authors are proposing a "novel" model about digital on-boarding finance that should mitigate the state-of-the-art cybersecurity risks. Further, the goals of the project consist to improve the user experience in the financial sector. Although the contribution is well written and well organized, I have the following concerns:

- How the SOTER project can be considered "different" compared to the current state of the art? There are several solutions and projects that are enabling the biometric-based digital authentication (I tried to search on Google: finance biometric authentication). Please, try to highlight better your novelty.
- How can the SOTER project be compliant to several regulatory frameworks if each of them propose a different methodology that should be applied to achieve the same goal? Are not they in contrast among them?
- Which biometric data are adopted? Further, which are the tools (hardware) that a user needs to access from home?
- It seems that Everis already implemented all of the features mentioned in the project. Basically, they integrated the existing solutions into one platform.
- When the authors say that the mobile application can be easily integrated in the entity's mobile phone, what do you mean? Did you develop a sort of built-in application feature for the operating system?
- It is an interesting anti-spoofing mechanism proposed, i.e. proof of live based on blinking and unpredictable movements. What happens if a malicious user train a system that can reproduce exactly on a screen the "unpredictable movements". A video-source can be adopted to trick the system? Try to have a look on https://www.synthesia.io/.
- How does Accertify provide the device identification? What are the technical details about the device fingerprinting?
- Put a reference for the Malware as a Service.
- For the SSl/TLS the inspection is done via Deep Packet Inspection. Please try to improve the description about the techniques that can be adopted.

Where applicable, are recommendations and next steps explained clearly for others to follow? (Please consider whether others in the research community would be able to implement guidelines or recommendations and/or constructively engage in the debate)

Partly

Does the article adequately reference differing views and opinions?

Yes

Are all factual statements correct, and are statements and arguments made adequately supported by citations?

Partly

Is the rationale for the Open Letter provided in sufficient detail? (Please consider whether existing challenges in the field are outlined clearly and whether the purpose of the letter is explained)

Partly

Is the Open Letter written in accessible language? (Please consider whether all subject-specific terms, concepts and abbreviations are explained)

Yes

Reviewer Expertise:

Unmanned Aerial Vehicles Security, Maritime Security, Wireless Security, Internet of Things (IoT), Applied Cryptography, Privacy Preserving Systems, Critical Infrastructure Security, and Cyber-Physical Systems Security

I confirm that I have read this submission and believe that I have an appropriate level of expertise to confirm that it is of an acceptable scientific standard, however I have significant reservations, as outlined above.

# Response

**Published:** 2022-02-08

**Authors:** Esther Aguilera

**Affiliations:** NTT DATA, Spain

- *them proposes a different methodology to be applied to achieve the same goal? Are they not in contrast with each other?*

Each regulatory framework is necessary for legislative compliance with the standards required for the different modules integrated in the process, so there is no confrontation between them.

- *Which biometric data are adopted? Further, which are the tools (hardware) that a user needs to access from home?*

Sorry, I don’t understand what you want to mean.

- *When the authors say that the mobile application can be easily integrated into the entity's cell phone, what do they mean? Have they developed a kind of application feature integrated into the operating system?*

No, what they are referring to here is that it is a modular solution, which can be easily integrated into the core banking of the entity, due to the architecture that has been used and the possibility of adding, removing, or replacing any of the modules that make up SOTER, for another that the bank wants.  

- *It is an interesting anti-spoofing mechanism proposed, i.e. proof of live based on blinking and unpredictable movements. What happens if a malicious user train a system that can reproduce exactly on a screen the "unpredictable movements". A video-source can be adopted to trick the system? Try to have a look on https://www.synthesia.io/*

You could not fool the system so easily, in fact if you put a video or a photo in front of the camera it detects that it is not a real person, because from checking that the face matches the one taken from the ID card, it is also able to identify the depth of the image it is capturing, so even if it was a video, it could not capture that depth and it would detect fraudulent behavior.

- *How does Accertify provide the device identification? What are the technical details about device fingerprinting?*

This information is confidential, Accertify doesn’t want to detail it in this document.

- *Put a reference for the Malware as a Service*

Do you mean something like this https://clario.co/blog/malware-as-a-service/#:~:text=Slack%20and%20Dropbox%20are%20examples,customers%20on%20a%20subscription%20basis.?

- *For the SSl/TLS the inspection is done via Deep Packet Inspection. Please try to improve the description about the techniques that can be adopted.*

I don’t understand what you want to say. Can you give me an example?
